# Supplementary figures and images for: The Initial Gut Microbiota and Response to Antibiotic Perturbation Influence Clostridioides difficile Clearance in Mice
Source: mSphere. 2020 Oct 21;5(5):e00869-20. doi: 10.1128/mSphere.00869-20 (PMC7580958; doi:10.1128/mSphere.00869-20)

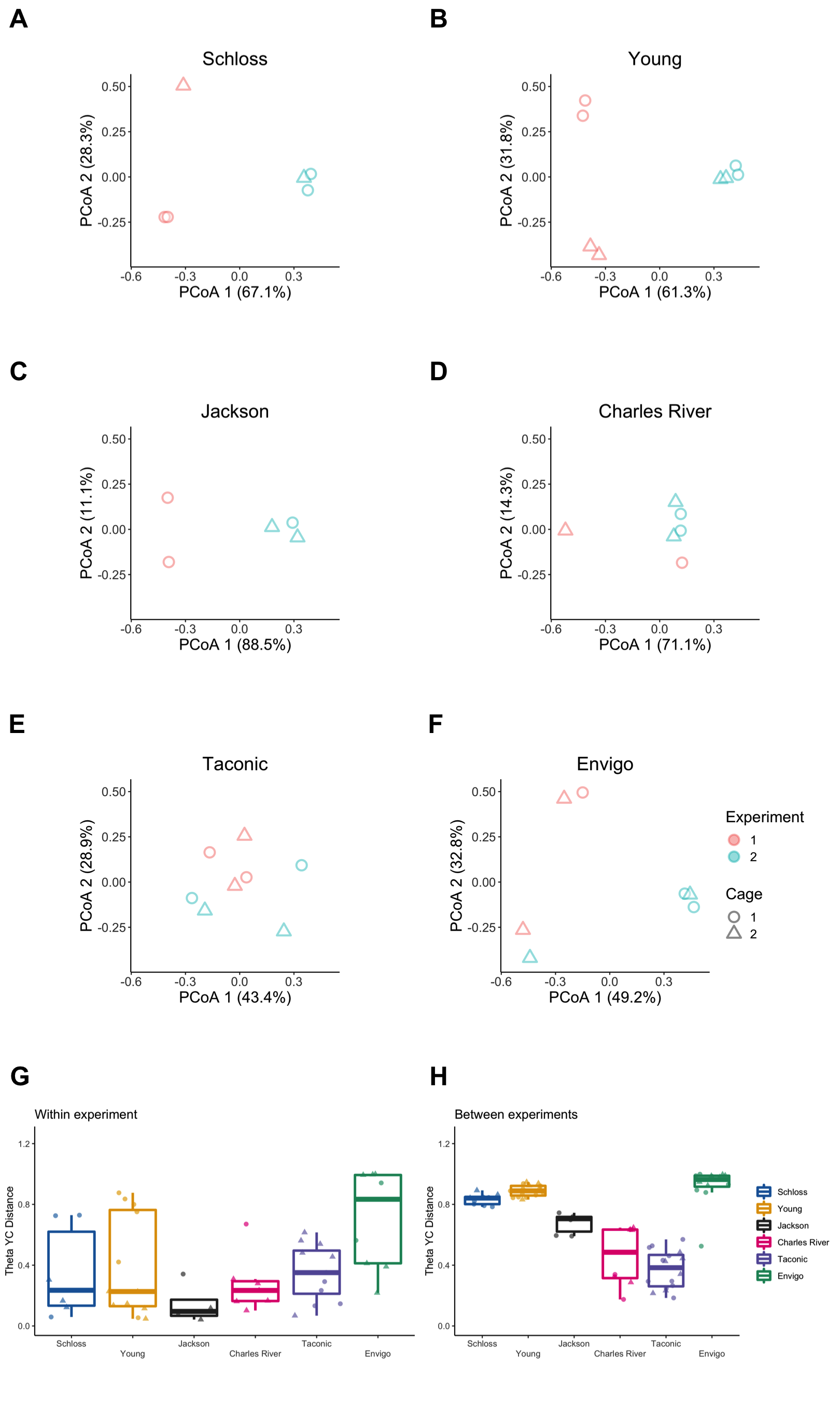

Supplement: FIG S1 [file mSphere.00869-20-sf001.tif]

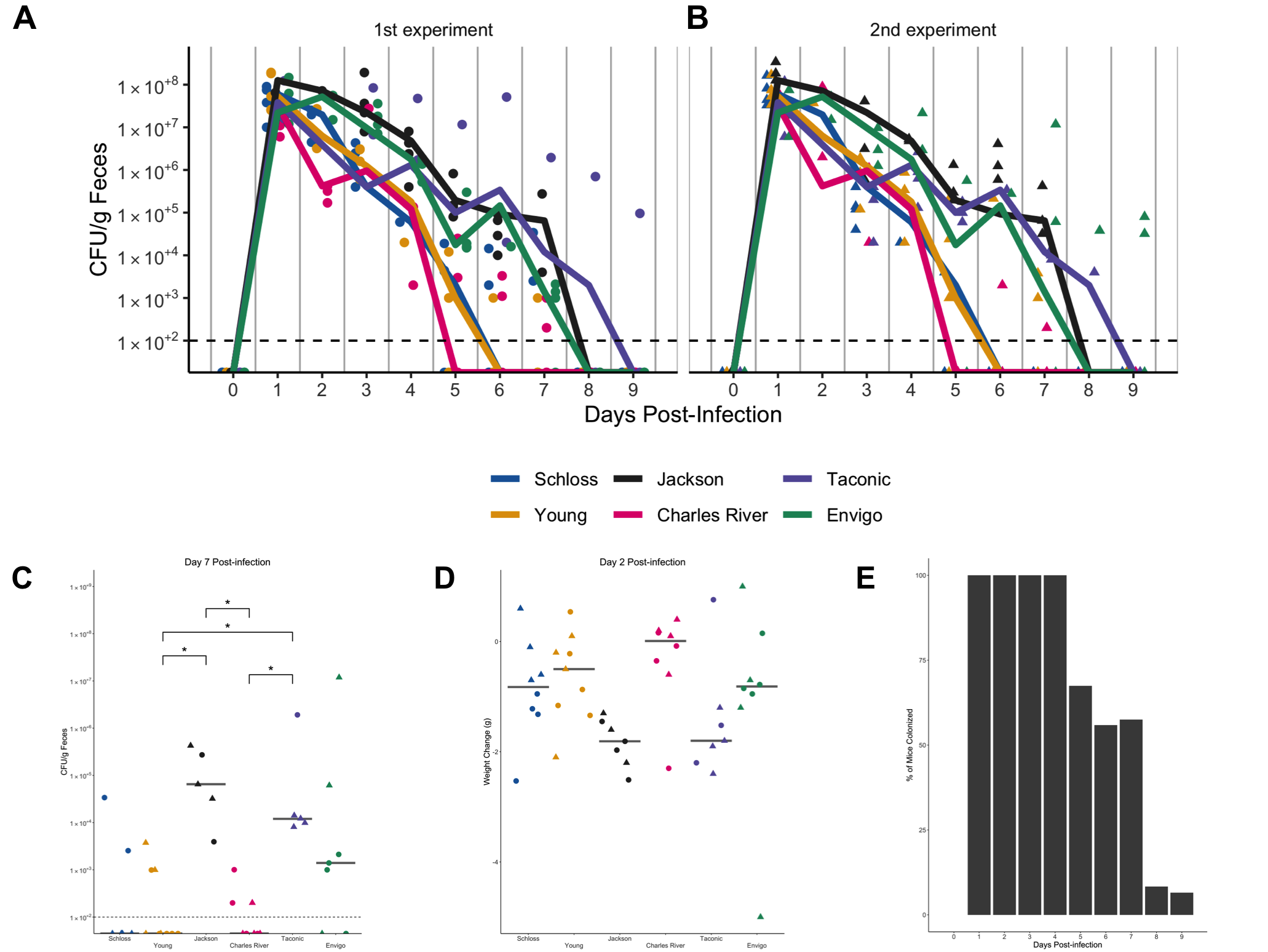

Supplement: FIG S2 [file mSphere.00869-20-sf002.tif]

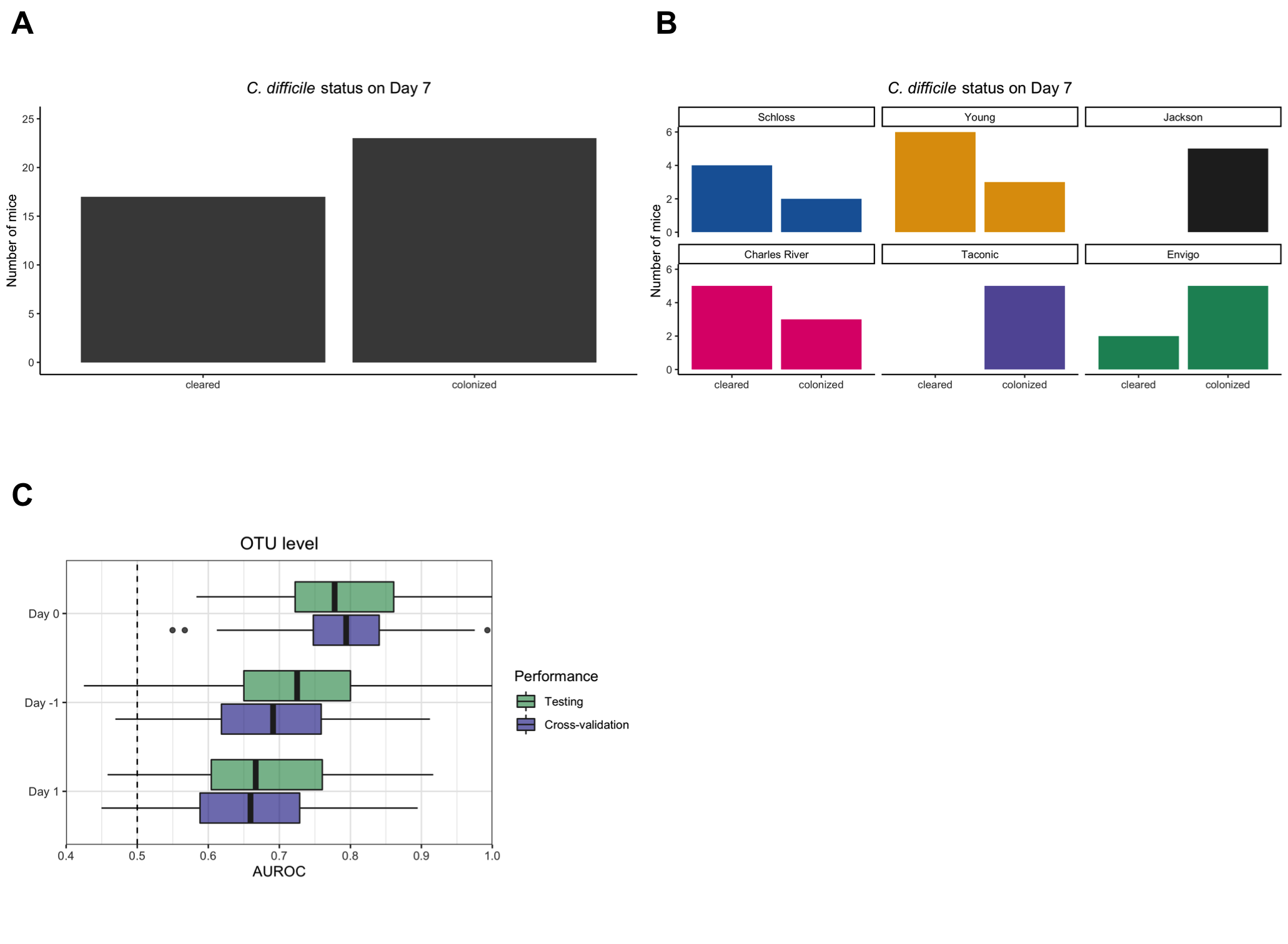

Supplement: FIG S3 [file mSphere.00869-20-sf003.tif]

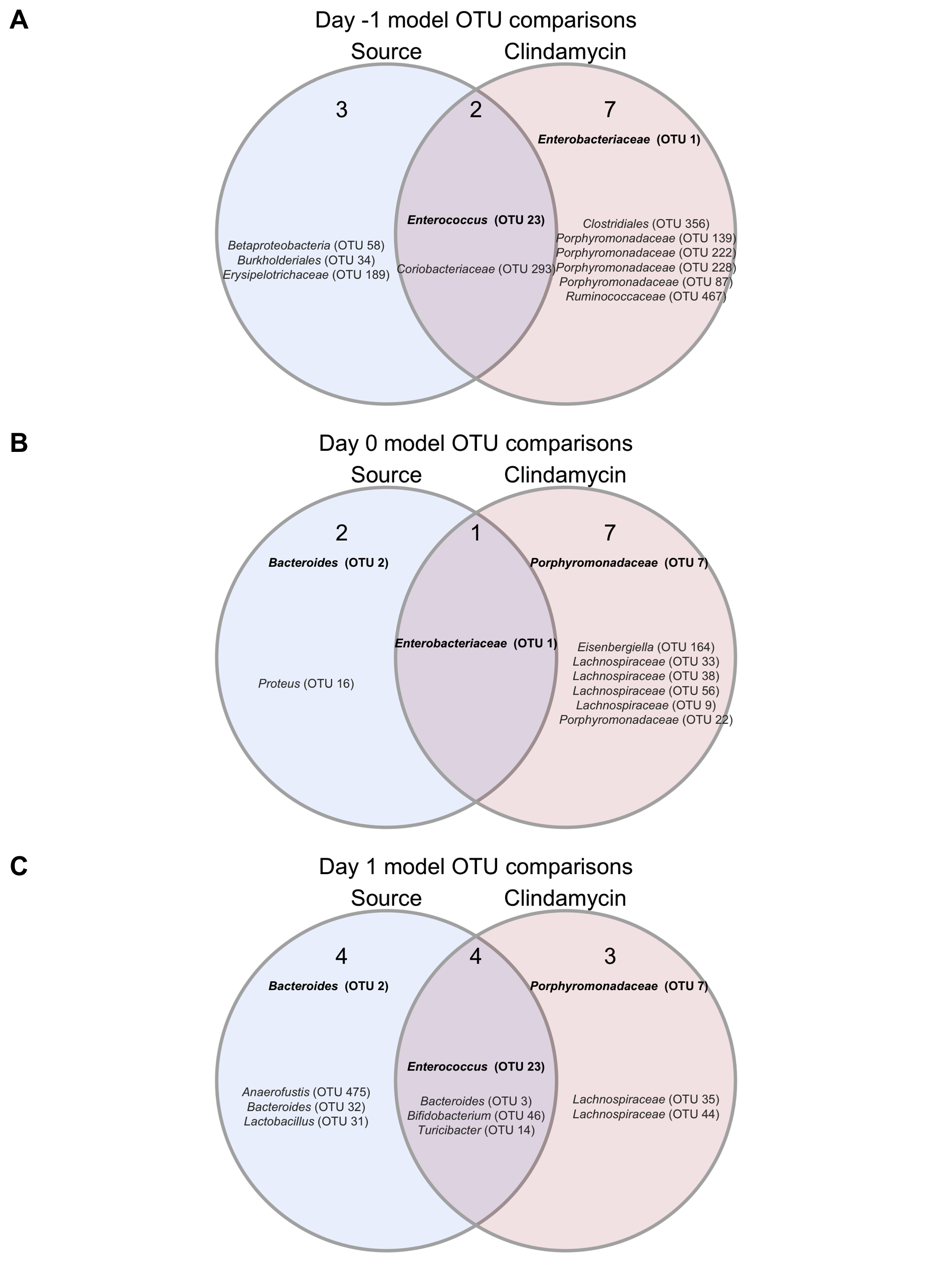

Supplement: FIG S4 [file mSphere.00869-20-sf004.tif]
